# Challenges and Technological Strategies to Enhance Probiotic Viability in Non-Dairy Food Matrices

**DOI:** 10.3390/molecules31152663

**Published:** 2026-07-30

**Authors:** Stevens Duarte, Janaina Sánchez-García, Ester Betoret, Noelia Betoret

**Affiliations:** 1 Instituto Universitario de Ingeniería de Alimentos—FoodUPV, Universitat Politècnica de València, Camino de Vera s/n, 46022 Valencia, Spain; steduase@upv.edu.es (S.D.); jmsanga1@upv.edu.es (J.S.-G.); mesbeval@upvnet.upv.es (E.B.); 2 Facultad de Ciencias de la Salud y Desarrollo Humano, Universidad Ecotec, Km. 13.5 Vía Samborondón, Samborondón 092302, Ecuador

**Keywords:** probiotics, non-dairy foods, food matrix, fermentation, probiotic viability, emerging technologies, encapsulation, stress adaptation, evolution

## Abstract

The growing demand for plant-based and functional foods has driven increasing interest in the incorporation of probiotics into non-dairy matrices. However, maintaining probiotic viability throughout processing, storage, and gastrointestinal transit remains a major challenge due to exposure to multiple environmental stresses. This review aims to provide a comprehensive overview of the incorporation of probiotics into non-dairy food systems, the key factors that affect their survival, and the available strategies to enhance their stability and functionality. First, the main approaches for incorporating probiotics into food matrices, including direct addition and fermentation processes, are discussed. Second, the critical factors influencing probiotic viability are examined, including intrinsic strain characteristics, the composition and physicochemical properties of the food matrix, and the impact of processing, storage, and gastrointestinal conditions. Third, current strategies to improve probiotic survival are analyzed, including the control of processing parameters and the use of emerging processing technologies, structural protection through micro- and nanoencapsulation, and biological approaches such as stress adaptation and strain improvement. Overall, the integration of technological and biological strategies provides a robust framework for enhancing probiotic stability in non-dairy foods. Future research should focus on optimizing these approaches while ensuring product quality and consumer acceptance, facilitating the development of effective and commercially viable functional foods.

## 1. Introduction

The term ‘functional foods’ refers to foods that provide nutrition and help prevent disease. These foods can be either naturally occurring or have bioactive compounds added during the manufacturing process [1]. Bioactive compounds such as probiotics contribute to host health by producing antimicrobial compounds, competing for adhesion sites and nutrients with other species, and stabilizing the intestinal microbiota [2].

Fermentation is the most widespread method of incorporating probiotics, although its primary purpose is to extend the shelf life or to manufacture specific foods [3]. It is a common method of incorporating probiotics in dairy products such as yoghurt and cheese, in beverages such as beer, kefir and kombucha, in meat products such as ham and fuet, as well as in vegetable products such as sauerkraut, kimchi and pickled gherkins. Generally, fermentation process involves a multitude of chemical, enzymatic and microbiological reactions, typically involving complex populations and/or communities of microorganisms [4]. Many of the bacteria or yeasts responsible for producing traditional fermented foods have been identified as having probiotic properties and include several genera such as *Bifidobacterium*, *Bacillus* and lactic acid bacteria (LAB) [5]. For example, *Lactobacillus delbrueckii* ssp. *bulgaricus* plays a significant role in the development of the organoleptic, hygienic and probiotic characteristics of yogurt and fermented milk [6]. Also, strains of *Levilactobacillus brevis*, *Lacticaseibacillus rhamnosus*, *Lactiplantibacillus plantarum* and *Lacticaseibacillus casei* with recognized probiotic properties are used for cabbage fermentation to obtain sauerkraut [7].

Alternatively, it is possible to develop probiotic food by incorporating one or more strains of probiotic microorganisms with a proven effect into a food matrix of interest, without undergoing fermentation. Enriching or fortifying food with probiotics is a well-established practice that increases the supply of probiotic foods. This procedure is of particular interest when working with non-dairy foods, as the growth and survival of probiotic bacteria such as LAB and *Bifidobacterium* may be compromised. The characteristics of the strain and the composition and properties of the food into which the probiotic cells are incorporated will be decisive factors in their viability throughout their shelf life and during gastrointestinal digestion. Garcia et al. [8] assessed the survival of five fruit-derived and freeze-dried strains of LAB, including species formerly classified within the genus *Lactobacillus*, when incorporated into apple, grape, and orange juices stored under refrigerated conditions. They inoculated 200 mL aliquots of fresh fruit juice samples with 2 g of freshly freeze-dried LAB, with final counts varying between 8–9 log CFU/mL. Stress tolerance, whether due to the acidity of the juices or to digestive conditions, varied among the LAB strains analyzed. All freeze-dried strains maintained high survival rates after refrigerated storage for up to 14 days in apple juice, and *Lactiplantibacillus plantarum* 49 and *Lacticaseibacillus paracasei* 108 also showed high survival rates in orange and grape juices. Although changes in the physicochemical properties of the juices were not significant, slight increases were observed in the content of organic acids and total soluble solids, which might be explained by metabolic reactions associated with the growth of the microorganisms or limited malolactic fermentation processes that some strains carry out as an adaptation to acidic conditions. The authors suggest that a brief adaptation phase prior to inoculation could improve the survival of the test strains when incorporated into the fruit juices analyzed.

In any case, regardless of the microbial species used or the food matrix considered when developing a probiotic food, according to the latest consensus on the term ‘probiotic’, the microorganisms will have to be able to exert a beneficial effect on the consumer’s health [9]. To achieve this, probiotic cells must reach the end of the human gastrointestinal tract in a viable state and at a certain concentration. They must therefore be able to withstand the processes involved in their production, such as incorporation, processing, distribution and storing, as well as preparation at home, ingestion and gastrointestinal digestion. The viability of any microbial cell is highly dependent on the nature and characteristics of the strain. Its origin determines the conditions to which it is most adapted, as do the characteristics of the food matrix to which it is added and the stress conditions to which it has been subjected during processing. In many cases, subjecting microbial cells to controlled stress conditions increases their resistance to other conditions. The strategies implemented to protect microbial cells are also important, and operations such as encapsulation can be decisive in ensuring viability throughout the gastrointestinal tract. This review examines the incorporation of probiotics into non-dairy food matrices, with emphasis on the main factors affecting microbial viability during processing, storage, and gastrointestinal digestion. It also critically evaluates technological and biological strategies designed to enhance probiotic survival and functional performance, including the control of processing conditions, structural protection systems, and strain-level adaptation strategies.

## 2. Incorporating Probiotics into Food Matrices

The successful delivery of probiotics into the target action human site relies on adequate strain selection and cultivation, suitable processing and compatible food matrices that maintain cell viability and bioactivity during production, storage, and gastrointestinal transit. Currently, probiotics can be incorporated into non-dairy food matrices through two main approaches: (i) fermentation of the food matrix itself using probiotic or potentially probiotic microorganisms, and (ii) direct incorporation of viable probiotic cells during food processing. Both approaches present specific technological challenges and advantages, which are discussed below.

### 2.1. Incorporation Through Fermentation

Food fermentation is the oldest technique for producing probiotic foods, whereby the food matrix itself acts as the substrate. It can be defined as a biochemical process in which microorganisms (bacteria, yeasts, fungi) convert food components (such as sugars, starches, proteins, lipids) into other compounds (organic acids, alcohols, gases, peptides, etc.) through their metabolism under controlled conditions. During this process, microbial enzymes also act to modify the structure of food components, generating changes in flavor, texture, nutritional value, safety, and shelf life [10]. Non-dairy fermented foods such as sausages, kimchi, kombucha, sour beers, miso and sourdough bread contain probiotic microorganisms and other compounds generated during fermentation, which give them their functional value [11]. In the food industry context, the objective is multiple: food preservation, increase food sensory value (aroma, flavor) and/or nutritional value (e.g., release of bioactive compounds), reduce antinutritional or toxic compounds, and provide microorganisms that are potentially beneficial to health. In relation to the last objective, in recent years and with the explosion of omics techniques, there has been increased interest in understanding how complex microbial communities and metabolites generated during food fermentation can modulate the gut microbiome and affect human health [12]. Depending on the desired outcome, it may be advisable to approach the fermentation process differently. In the case of traditional spontaneous fermentation, the native microbiota, usually better adapted to fermentation processes, is responsible for the process without the intentional addition of defined starter cultures. Autochthonous microbes often exhibit probiotic potential, including the ability to survive gastrointestinal transit, modulate the host immune response, and compete against pathogens [13]. However, the evolution of the microbial population throughout the process can be highly variable. Therefore, evaluating the microbiota in the final product is necessary to ensure its quality and safety [4]. In some cases, a controlled batch fermentation with the addition of starter cultures may be necessary to produce a probiotic food with a specific microbial species, or to ensure that the final product has specific physical, chemical and organoleptic properties. By this means, it is possible to choose starter cultures that are already recognized as probiotics, control conditions to optimize their growth, and obtain a final product with previously defined characteristics [14]. In immobilized cell fermentation, probiotic cells are attached to a solid carrier that releases them gradually into the food. This method is particularly beneficial for producing liquid fermented foods with an improved shelf life and targeted administration. Additionally, immobilized cell systems enable the repeated or continuous use of probiotic cultures, thereby improving process efficiency and reducing production costs [15]. When starter cultures are used, competition for metabolites, as well as antagonistic or synergistic relations, can be established between the autochthonous microbiota and the probiotic starters. Antagonistic effects have been mainly linked to the secretion of inhibitory substances as bacteriocins [16]. Synergistic effects are primarily related to metabolic complementation, whereby the metabolites produced by one microorganism serve as a substrate for another [17]. For example, this occurs between yeasts and lactic acid bacteria during the fermentation of bread, wine, and dairy products [18]. Also, cooperative interactions between yeasts such as *Brettanomyces bruxellensis* and bacteria such as *Komagataeibacter intermedius* contribute to the successful fermentation of kombucha [19]. In addition, the production of growth factors, including vitamins and amino acids, has been shown to stimulate the proliferation of associated microorganisms in mixed fermentations [20]. Similarly, in vegetable fermentations such as white cabbage, the use of probiotic starter cultures has been reported to significantly reduce antinutritional compounds, including phytates, tannins, and oxalates, by 42–66%, depending on the compound and processing conditions, compared to spontaneous fermentation [21]. In these cases, it can be advantageous to carry out co-fermentation and develop an inoculation strategy to enhance the efficiency of the process, while considering the functional compatibility of the selected strains [22]. In symbiotic fermentation, both microbial cells and prebiotics are added at the same time. Prebiotics are non-digestible substances that can be utilized by microbial cells, thereby increasing their viability and stability [23].

To better understand and optimize fermentation processes, advances in metabolomic, proteomic, transcriptomic, and sequencing technologies have provided a more detailed understanding of the microbial ecology of fermented foods, enabling the generation of models of microbial interaction networks [24]. Even though these efforts are based on theory, they can still lead to important results that are useful in practice. For example, they can be used to predict the quality of wine based on the microbes present in the grapes at the time of harvest. They can also be used to predict the sensory characteristics of kimchi, such as its flavor and aroma, based on the origin of its ingredients [25].

### 2.2. Direct Incorporation of Probiotic Cells

Another way to obtain probiotic foods is to incorporate viable microbial cells directly into food during processing. This is similar to an enrichment process. However, changes to the product’s sensory profile will undoubtedly occur, and these must be accepted by consumers, the process of enrichment does not alter the original characteristics of the food to any great extent [26]. Prior to incorporation into the food matrix, probiotic biomass must be produced under controlled cultivation conditions. Achieving high yields of probiotic biomass requires employing production procedures that are economically viable and optimizing the substrate used and the cultivation conditions such as the pH level and the incubation time [27]. Specific studies quantified these effects in *Lactiplantibacillus plantarum* 200655 by optimizing the medium composition with maltose, yeast extract, and soytone; the biomass yield reached 3.951 g/L, compared to 2.429 g/L with the unoptimized medium. When this optimized medium was applied in a bioreactor under ideal conditions (30 °C, pH 6.5, and agitation at 200 rpm), the biomass reached 5.866 g/L after 18 h [28]. Research has been conducted on probiotic enrichment of fruit juices, vegetable drinks, breakfast cereals and snacks [29]. In these cases, it should be noted that probiotic viability will depend greatly on the food matrix into which they are incorporated, and protection strategies such as microencapsulation or coating. Strain selection will almost always be required to maintain viability throughout the food’s shelf life. Specifically, Barik et al. [30] demonstrated that, in the case of fruit juices, survival depended heavily on the juice’s pH, storage temperature and the use of microencapsulation. With microencapsulation, it was possible to maintain viable counts for weeks. Ogidi et al. [31] evaluated the viability of lactic acid bacteria in formulated plant-based beverages enriched with extracts from selected edible plants namely, date fruits, mustard seed, and turmeric rhizome. The researchers revealed the survivability of probiotics such as *Lacticaseibacillus rhamnosus*, *Lactiplantibacillus plantarum*, *Lactobacillus acidophilus*, and *Lacticaseibacillus casei* in plant-based beverages produced from coconut, oat, rice, tiger nut, soybean, almond, and others. The incorporation of extract from date fruit, mustard seeds, and turmeric into plant milk supported the growth of *Lactobacillus acidophilus*, *Limosilactobacillus fermentum*, and *Lactiplantibacillus pentosus* for four days, although it would be necessary to study the feasibility for longer periods of time. Numerous studies have been conducted to investigate the best way to incorporate probiotics in cereal based baked foods [32]. Microencapsulated probiotics, edible films containing probiotics, spore-forming bacteria, and probiotic addition after baking are the main strategies to formulate probiotic cereal based baked foods. Mani-López et al. [32] concluded adding probiotics after baking into through covers or fillings is an interesting option without concerns about probiotic viability.

The selection of the most appropriate strategy depends on the characteristics of the food matrix and the intended properties of the final product. Fermentation is generally preferred for foods in which microbial metabolism contributes to the development of desirable sensory, technological and nutritional characteristics. In contrast, direct probiotic incorporation is more suitable for products in which the original characteristics should be largely preserved while providing probiotic functionality.

**Figure 1:**
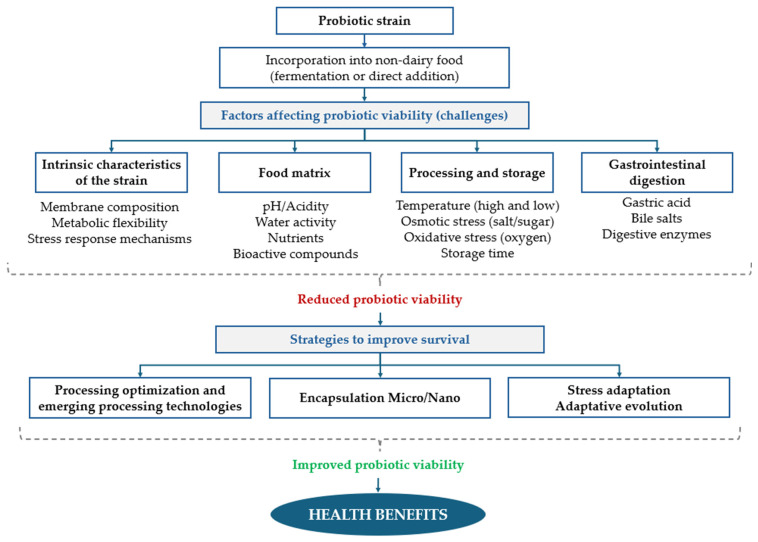
Overview covering the incorporation of probiotic into non-dairy food matrices, the main factors affecting viability, and technological strategies to improve survival.

To facilitate the understanding of the different factors influencing probiotic survival and the technological approaches discussed throughout this review, Figure 1 provides an integrated overview of the relationships between non-dairy food matrices, the major environmental challenges affecting probiotic viability, and the principal strategies currently available to improve microbial survival and functionality.

## 3. Factors Affecting the Viability of Probiotics in Non-Dairy Matrices

### 3.1. Intrinsic Characteristics of the Strain

The viability of probiotics in non-dairy foods depends largely on the strain selected. Each strain has unique biological traits that influence its survival under stress conditions typical of non-dairy matrices. Commonly used strains belong to genera such as *Bacillus* and *Bifidobacterium*, as well as lactic acid bacteria (LAB) and some yeasts like *Saccharomyces*, which have shown documented probiotic potential [33,34,35]. Traditional fermented foods often contain undefined microbial populations, making it difficult to identify the probiotic strains present at consumption. Therefore, knowing the identity and suitability of the inoculated microorganisms is essential before using them in any fermentation process [36]. Intrinsic microbial traits affecting viability include the ability to tolerate environmental stressors such as acidity, osmotic pressure, oxidative conditions, temperature fluctuations and exposure to oxygen, all of which microorganisms frequently encounter during food production and storage [37]. Compared with dairy systems, plant-based substrates often exhibit lower nutrient availability, unfavorable pH, antinutritional factors, and reduced buffering capacity, conditions that can compromise probiotic survival and make intrinsic stress resistance particularly critical [38].

The tolerance of microbial strains to environmental stressors in food is shaped by physiological features such as membrane composition and metabolic flexibility, as well as by protective mechanisms that are activated in response to stress [37]. Probiotic microorganisms adapt to environmental stress through modifications in cell morphology and membrane fatty acid composition [39,40,41], supported by conserved stress-response proteins and regulatory systems whose effectiveness varies between species. Additional mechanisms—such as ATP-driven proton extrusion, amino-acid decarboxylation pathways that stabilize intracellular pH, membrane-associated chaperones and DNA-repair systems—further reinforce survival under adverse conditions, highlighting the multifaceted and species-specific nature of probiotic stress adaptation [39]. These strategies and mechanisms are described in greater detail in Table 1.

Building on these mechanisms, experimental evidence from different probiotic strains illustrates how such physiological strategies translate into measurable improvements in stress tolerance. For example, *Lactiplantibacillus plantarum* KLDS 1.0328, cultured under controlled conditions, exhibited clear physiological adjustments under osmotic, acidic and alkaline stress, including stress-induced modifications in membrane fatty acid composition—reflected in increased unsaturated-to-saturated ratios under all treatments except alkaline pH—and marked changes in surface properties. Under 6% NaCl, growth was severely inhibited and cell numbers fell to 5.39 × 10^8^ CFU/mL after 24 h, accompanied by reduced hydrophobicity and aggregation, whereas pH 5.0 and pH 8.0 supported robust proliferation [41]. Moreover, the physiological state of the cells at the time of inoculation—such as being in exponential phase—can enhance stress tolerance, particularly under optimized fermentation conditions [37]. In *Lentilactobacillus buchneri* R1102 and *Bifidobacterium longum* R0175, both cultivated under controlled fermentation conditions, harvesting time strongly influenced membrane characteristics and freezing tolerance. Exponential-phase cells had a higher unsaturated-to-saturated fatty acid ratio—1.9 times greater in *L. buchneri* and 3.7 times greater in *B. longum*—than stationary-phase cells. Exponential-phase cells also exhibited greater membrane fluidity, which is essential for cell division, whereas stationary-phase cells became more rigid due to stress-related changes in fatty acid composition. Viability after freezing varied between species: *B. longum* survived better in the stationary phase, whereas *L. buchneri* showed similar survival at both stages. Acidification activity, used as a functional indicator of resistance to freezing, was higher in exponential-phase *L. buchneri* but showed no major differences between growth phases in *B. longum* [42].

**Table 1:** Cellular strategies and mechanisms employed by probiotic bacteria to withstand environmental stresses. Adapted from Lillo-Pérez et al. [38] and Ruiz et al. [43].

Stress Condition	Typical Source in Food Systems	Strategy and Mechanism of Response
Heat	High drying temperatures	Maintenance of protein homeostasis: Proper protein folding by molecular chaperones. Degradation of misfolded proteins by proteases. Regulatory network through transcriptional regulators.
Cold	Freeze-drying; low storage temperature	Cold adaptation response: Small heat shock proteins. Cold shock proteins. Cold-induced proteins.
Acid	Organic acids during cultivation; acidified carrier products; gastric conditions	Intracellular pH regulation: F_1_F_0_-ATPase-mediated proton extrusion. Cytoplasmic buffering or ammonia production from branched-chain amino acids and glutamine synthetase. Cell wall modification by esterases.
Oxidative	Oxygen exposure during fermentation, drying, storage, and consumption	Detoxification of reactive species: Reactive oxygen species (ROS)-scavenging enzymes. Protective proteins targeting RNA and DNA. Proteins involved in shielding cells from oxidative stress.
Bile salts	Bile salts in the small intestine	Detoxification and surface adaptation: Bile salt/acid detoxification by multidrug transporters and bile efflux pumps (e.g., BetA, Ctr). Bile salt deconjugation by bile salt hydrolase. Alteration of cell surface: extracellular exopolysaccharide production, changes in fatty-acid composition, and modifications in surface-associated proteins. Changes in energy metabolism through shifts in ATP synthesis and glycolytic end-products. Modification of redox-related enzymes. Proper protein folding by molecular chaperones. Degradation of misfolded proteins by proteases.
Antimicrobial compounds	Presence of inhibitory substances in food matrices	Cytoplasmic detoxification: Detoxification by efflux pumps.
Carbon source fluctuations	Variable carbohydrate availability in plant matrices	Metabolic versatility: Ability to degrade a wide range of carbohydrates through extracellular oligosaccharide-binding proteins, glycosidases and transcriptional regulators.

Another protective response activated under stress is the synthesis of exopolysaccharides (EPSs), a capability found in several Gram-positive and Gram-negative microorganisms. Among them, the genera *Lactobacillus* and *Bifidobacterium* have clear probiotic relevance and are the most extensively studied. EPSs are high-molecular-weight extracellular carbohydrates produced in response to stressors such as pH, temperature and osmotic shifts, and they support microbial survival by enhancing adhesion and protecting cells against biotic and abiotic stress [44]. Experimental evidence shows that EPS synthesis contributes to the intrinsic ability of probiotic strains to maintain viability under changing environmental conditions. In *Bifidobacterium animalis* subsp. *lactis* SF, cultivated in BS medium under anaerobic fermentation at 37 °C (0–36 h), EPS production increased in parallel with cell growth, reaching 300 mg/L as viable counts rose to 9.47 log CFU/mL at pH 3.0 [45]. In *Lactiplantibacillus plantarum* HMX2, fermented in MRS medium at 37 °C for 14 h under controlled pH conditions, cells maintained clear growth and viability at pH 4.5, while EPS synthesis remained active and increased per dry cell weight during the stationary phase [46]. Likewise, for *Lactiplantibacillus plantarum* VAL6, cultivated in MRS medium and exposed to acidic (pH 3), alkaline (pH 8) and osmotic (10% NaCl) stress, the strain maintained viability above 5 log CFU/mL, while EPS production increased under these conditions, reaching a highest yield of 50.44 g/L [47].

On the other hand, the development of new probiotic strains capable of surviving and remaining functionally active in non-dairy food matrices is becoming increasingly important. Creating robust strains is essential for formulating functional foods that meet diverse dietary needs. Uhegwu & Anumudu [48] provide a conceptual comparison between conventional probiotic species, such as LAB and *Bifidobacterium*, and emerging candidates including *Akkermansia muciniphila* and *Faecalibacterium prausnitzii*. While conventional strains from traditional fermented foods typically exhibit antimicrobial activity, acid and bile tolerance, and epithelial adhesion, emerging strains derived from human or marine gut microbiota, soil or plants are distinguished by their immunomodulatory capacity and production of short-chain fatty acids [48]. However, each group presents specific challenges: conventional species show strain-dependent variability and potential genetic instability under industrial conditions, whereas emerging strains face difficulties related to cultivation and stability within food matrices [48].

Finally, intrinsic microbial traits related to stress tolerance are not fixed but can be strengthened through adaptation. When microorganisms are gradually exposed to stressful environments, they undergo modifications that enhance their ability to withstand subsequent stress, a process supported by mechanisms such as long-retention proteins, epigenetic regulation and cross-protection across different stress conditions. These adaptive responses improve the capacity of strains to cope with environmental challenges and contribute to the development of more resilient phenotypes [49].

### 3.2. Food Matrix: Composition and Physicochemical Properties

The food matrix plays a critical role in the successful delivery, viability and functionality of probiotics. Components such as fermentable carbohydrates, dietary fiber, lipids, proteins and bioactive compounds can either protect or enhance the activity of probiotic microorganisms, which is crucial for their survival [50,51]. Besides their nutritional composition, plant-based food matrices contain phenolic compounds that may represent an additional chemical challenge for probiotic microorganisms [52]. Depending on their concentration and the environmental conditions, these compounds can alter cell-envelope properties, modify membrane integrity and protein activity, and consequently influence bacterial growth and survival [52]. However, these effects are highly strain-dependent, since some *Lactobacillus* strains are able to metabolize plant phenolic compounds and activate adaptive responses involving the remodeling of cell-envelope components, including peptidoglycan, teichoic acids, surface polysaccharides and surface proteins, thereby improving their fitness in plant-based environments [52,53].

Carbohydrate composition plays an important role in probiotic performance. Ref. [54] showed that, in soymilk supplemented with 10 g/L fructooligosaccharides (FOS), inulin, mannitol, maltodextrin or pectin and fermented at 37 °C for 24 h, all probiotic strains tested (*Lactobacillus* sp. FTDC 2113, *Lactobacillus acidophilus* FTDC 8033, *Lactobacillus acidophilus* ATCC 4356, *Lacticaseibacillus casei* ATCC 393, *Bifidobacterium* FTDC 8943 and *Bifidobacterium longum* FTDC 8643) reached viable counts between 7.08 and 9.10 log CFU/mL. Growth enhancement was strain-dependent, with the largest increase observed in *L. acidophilus* FTDC 8033, where FOS and mannitol increased growth by 4.30% and 3.95% compared with the control. Prebiotics also stimulated lactic acid production and increased α-galactosidase activity, facilitating the hydrolysis of soy oligosaccharides and the utilization of simpler sugars such as fructose and glucose, thereby supporting microbial metabolism during fermentation [54].

Dietary fibers can also enhance probiotic activity by supplying fermentable polysaccharides that serve as prebiotic substrates for bacterial metabolism. Using dietary fiber fractions derived from a model plant-based snack, Kruk et al. [55] demonstrated that oat fiber and apple pomace stimulated the growth of *Lacticaseibacillus rhamnosus* ATCC 53103 compared with a control formulation. This effect was attributed to the presence of β-glucans and pectins, which were metabolized after the depletion of readily available sugars. Furthermore, combining both fiber sources prolonged the logarithmic or stationary growth phases, suggesting a synergistic prebiotic effect that sustained bacterial activity within the plant-based matrix. In another study, various plant-based food substrates were coated with *Bifidobacterium longum* ATCC15707ᵀ, *Lactobacillus acidophilus* ATCC4356ᵀ and *Lactiplantibacillus plantarum* RC30 to evaluate probiotic survival during storage. Wheat bran and oat provided greater stability than raisin, rice collet, coconut and peanut when stored at 20 °C and 20% relative humidity. After two weeks, *B. longum* exhibited no significant reduction in viability on wheat bran or oat, whereas both *L. acidophilus* and *L. plantarum* showed smaller declines on these substrates compared with the more pronounced reductions observed on the other matrices. *L. plantarum* stabilized after the initial decrease, with oat supporting the highest survival [56]. A similar pattern was observed when *Lacticaseibacillus casei* LC-1 was adhered and assessed in inulin, apple fiber, oat bran and green banana flour, identifying the latter two as the most effective matrices. These fibers showed the highest post-drying survival (79% and 76%), and SEM images confirmed uniform adhesion of *L. casei* LC-1 to their starch structures without morphological damage, together with the presence of exopolysaccharides (EPS). In this study, EPS production was interpreted as a protective response to dehydration stress that also favored adhesion to the fiber matrix. The moderate pH (≈5.8), low water activity (0.25–0.30) and the presence of β-glucans in oat bran and resistant starch in green banana flour further contributed to this protective microenvironment [57].

Lipid-rich matrices can protect probiotics mainly by reducing water availability and maintaining low-moisture environments, which limits cellular deterioration during storage. Water activity has been identified as a critical parameter governing probiotic stability in dry food systems [58]. This effect was demonstrated by Endo et al. [59], who reported that freeze-dried *Lacticaseibacillus rhamnosus* GG suspended in different oils maintained high viability during heat stress and four months of storage. In another study evaluating plant-based snack matrices, the composition of the formulation strongly influenced probiotic survival through differences in fiber content, lipid concentration and water activity. *Lacticaseibacillus rhamnosus* ATCC 53103 were directly inoculated into the base formulation—rich in insoluble fiber from dates, dried apples, peanut butter, oat fiber and apple pomace—forming a dense matrix with an intermediate aw of 0.53 ± 0.02, which led to complete inactivation after two months at 20 °C. In contrast, filled variants incorporated an additional peanut-butter core with markedly lower aw (0.27 ± 0.01), creating a lipid-rich microenvironment that preserved viability above 6 log CFU/g for up to five months at 20 °C [55]. A comparable protective effect was observed when *Lacticaseibacillus rhamnosus* GG was directly inoculated into peanut butter, where both full-fat (50.1%) and reduced-fat (39.9%) versions exhibited low water activity (aw ≈ 0.44) and near-neutral pH (6.10–6.35), conditions far more favorable for probiotic stability than the acidic range (pH 4.0–5.0) where viability typically declines. Under these physicochemical conditions, both matrices maintained *L. rhamnosus* GG above 6 log CFU/g at 4 °C, with no differences in viability between fat levels [60].

Protein-rich matrices also demonstrated strong protective effects. In soy-based cream cheese fermented by direct inoculation of *Lactobacillus acidophilus* FTCC 0291, the protein-rich matrix enabled the strain to remain above 10^7^ CFU/g for 20 days at both 4 and 25 °C. The strain utilized available reducing sugars and hydrolyzed soy oligosaccharides, while proteolysis released nitrogen-rich compounds in the form of peptides and amino acids. These metabolites supported bacterial growth and contributed to the formation of ACE-inhibitory bioactive fractions [61]. A similar behavior was observed in soy yoghurt fermented at 42 °C until pH 4.50/4.55/4.60, where mixed probiotic cultures including *Lactobacillus acidophilus* LAFTI L10, *Bifidobacterium animalis* subsp. *lactis* LAFTI B94, and *Lacticaseibacillus paracasei* LAFTI L26 maintained viability at about 8 log CFU/g over 28 days at 4 °C. Enhanced proteolysis increased free amino acids and peptides, supporting probiotic growth and contributing to ACE-inhibitory activity [62]. By contrast, in a study comparing cow’s milk with an oat-based beverage enriched with whey, pea or soy protein isolates, fermented at 37 °C until pH 4.60 and stored 7 days at 5 °C, the response of probiotics differed markedly between matrices. Although adding up to 3% protein isolates to both matrices promoted the growth of *Lacticaseibacillus casei* and *Lactobacillus johnsonii* before digestion (9–11 log CFU/g), their survival diverged sharply during simulated digestion. At the end of the intestinal phase, milks remained > 6.3 log CFU/g of *L. casei* and >7.2 log CFU/g of *L. johnsonii*, whereas oat beverages remained below 5 log CFU/g. In this case, the buffering properties and composition of milk proteins provided superior protection compared with the oat matrix. This shows that the overall composition of the food matrix—rather than protein addition alone—ultimately determines probiotic support [63].

Matrices rich in polyphenols can also sustain probiotic viability. In soy and almond milk fermented with *Lacticaseibacillus rhamnosus* ATCC 7469, *Lactobacillus acidophilus* ATCC 4356, *Lactiplantibacillus plantarum* ATCC 14917 and *Lacticaseibacillus casei* ATCC 393 at 40 °C for 9 h, viable counts remained within 6–7 log CFU/mL during 21 days of storage at 4 °C. Throughout fermentation and storage, total phenolics, flavonoids and antioxidant activity increased. The authors note that fiber, protein and polyphenols in these matrices may act as substrates supporting microbial growth during fermentation [64]. A similar trend was observed in wheat-bran beverages combined with red beetroot or carrots and fermented for 24 h at 28–37 °C with *Lactiplantibacillus plantarum* BR9, *Lactiplantibacillus plantarum* P35 and *Lactobacillus acidophilus* IBB801. Cell counts reached 8–9 log CFU/mL at the end of fermentation and remained at 7.5–8.9 log CFU/mL after one week at 4 °C, while free phenolics, flavonoids and DPPH scavenging activity increased markedly [65]. In honey-based kefir beverages fermented at 30 °C for 24 h and stored for 35 days at 5 °C, total phenolic compounds increased during fermentation, while LAB viability remained stable at approximately 10^7^ CFU/mL [66].

Finally, Erkmen & Bozoglu [67] stated that, in general, organic acids such as lactic, acetic, formic, and benzoic acids influence microbial viability because they are frequently present in their undissociated form, which facilitates their diffusion across the cell membrane. Once inside the cell, these acids dissociate and release protons (H^+^), leading to intracellular acidification and accumulation of acid anions. Consequently, cells must expend additional energy to maintain intracellular pH homeostasis, resulting in metabolic disturbances that ultimately suppress microbial growth [67,68].

### 3.3. Processing and Storage

Multiple scientific research studies show it is possible to incorporate very high initial levels of probiotic microorganisms, typically around 8–9 log CFU/mL or g, into non-dairy food matrices and retain these levels in products such as juices, plant-based beverages or other non-dairy functional foods [69,70,71]. *Lactiplantibacillus plantarum* subsp. *plantarum*, *Lacticaseibacillus rhamnosus*, *Lactobacillus acidophilus*, *Bifidobacterium animalis* and *Saccharomyces boulardii* were the species most commonly studied. The levels of probiotic microorganisms achieved in fruit and vegetable beverages were reviewed by Lillo-Pérez et al. [38] and Rasika et al. [72]. The fruit sources used as probiotic substrates included pomegranate, orange, banana, lemon, tomato, watermelon, apricot, cherry, passion fruit and pineapple. The nutritional value of the fruits, together with the possibility of easily modifying the composition of the beverages, ensured adequate levels of microorganisms after different storage periods. Remarkable values of *Lactiplantibacillus plantarum* subsp. *plantarum* were reported in pomegranate after 28 days of storage (10 log CFU/mL) [73] and in pineapple juice (10 log CFU/mL) [74]. Other plant-based beverages investigated included grain-based drinks from oat and rice, legume-based drinks from chickpea or soy, and nut-based drinks from almond, peanut or hazelnut. Levels of probiotic microorganisms between 6–8 log CFU/mL were achieved in most cases. Values between 5.5 and 6.5 log CFU/mL, of a mixed inoculum of lactic acid bacteria (LAB), originally described as *Lactobacillus*, were found in guava and acerola fermented by-products [75].

However, maintaining these high counts is challenging, as viability begins to decline even before consumption. During processing and storage, probiotic viability decreases as cells encounter multiple stress factors. Food-related parameters such as pH, acidity, oxygen, water activity, salt and sugar, together with heat treatment, freezing, thawing and drying, can damage membranes and disrupt metabolic activity. The storage environment, including temperature, moisture content, relative humidity, oxygen levels and light exposure, further affects stability, and packaging materials also influence these losses, making it difficult to maintain high viable counts throughout shelf life [76].

The main stresses suffered by microorganisms during processing and storage are thermal, osmotic and oxidative. The effect of processing is highly strain-dependent. Changes in the cell membrane and damage to DNA and proteins determine cell viability. High temperatures above 50 °C during drying, baking or cooking processes affect microbial activity and growth by altering the fatty acids located in the membrane. Likewise, at high temperatures, protein denaturation occurs due to the disruption of non-covalent interactions, which subsequently produces protein aggregation and damage to ribosomes and RNA.

Mani-López et al. [32] reviewed studies on the incorporation of probiotic microorganisms into cereal-based baked foods. The high temperatures reached during baking made the presence of viable probiotic microorganisms challenging. As an exception, *Lactiplantibacillus plantarum* subsp. *plantarum* cells were reduced to 6.7 log CFU/g in cupcakes [77] and to 4–5 log CFU/g in bread pieces with skimmed milk after baking at 200 °C for 8–10 min [78]. However, *Lactobacillus acidophilus* LA-5 did not exceed 3 log CFU/g in bread pieces with skimmed milk and butter [79], and levels of 2 log CFU/g of *Bifidobacterium animalis* were reached in a loaf of white bread after baking at 180 °C [80].

Conversely, exposure to low temperatures during freezing and storage decreases microorganism metabolism, inhibiting their growth [81,82]. Loss of cell turgor, changes in solute concentration and changes in cell volume can result from osmotic stress during drying, fermentation, salting or concentration [83]. However, differences have been reported between the effect of salt and sugar as well as in the ability of microorganisms to overcome the stress generated [84]. Exposure to oxygen during hot-air drying, spray drying or mixing can form reactive oxygen species, which may cause damage by reacting with proteins, lipids and DNA [82,85]. Furthermore, in dried cells, oxidative stress can occur due to the oxidation of cellular components, such as membrane lipid oxidation [86].

Despite the high initial probiotic counts achieved in many non-dairy foods, processing and storage conditions progressively reduce cell viability. Once consumed, probiotics must overcome an additional series of physiological stresses during gastrointestinal transit.

### 3.4. Gastrointestinal Digestion

Besides the intrinsic resistance of probiotic strains, the characteristics of the food matrix play a key role during gastrointestinal transit by modulating the exposure of microorganisms to gastric and intestinal stresses. During passage through the gastrointestinal tract, probiotics are exposed to adverse conditions, including highly acidic gastric pH, bile salts, digestive enzymes and other stress factors, which compromise their survival [87]. Evidence from in vitro digestion studies shows that, when probiotics are assessed without protective carriers, viability decreases sharply, with reductions of 2–4 log units frequently reported and final counts often falling below 6 log CFU/mL or g [87,88]. Exposure to acidic environments generally results in damage to the cell membrane, DNA and proteins [89]. Resistance to acidic conditions is crucial for probiotics to be able to exert their positive effects on health [2].

Various studies have shown that the composition and complexity of the food matrix play a decisive role in the resistance of probiotic microorganisms to the adverse conditions of gastrointestinal digestion. Solid matrices that are complex nutritionally and structurally provide greater protection against simulated conditions compared to simple matrices. This increased protection is due to a higher buffering effect, slower gastric emptying and the formation of physical microenvironments that minimize the direct exposure of probiotic cells to acidic pH and bile salts [90].

Naissinger da Silva et al. [88] evaluated the probiotic viability of eleven commercial microorganisms, supplied either as suspensions or in pill form, after simulated gastrointestinal digestion. All samples showed a reduction in viable counts, and only six maintained concentrations above 6 log CFU/g in the small intestine. Similarly, Islam et al. [91] compared the effect of simulated gastrointestinal digestion on free cells of *Lactobacillus acidophilus* and cells incorporated into a chocolate-based matrix. After digestion, levels of approximately 4 log CFU/g were detected in the chocolate matrix, whereas no viable cells were recovered from the free-cell suspension.

Matouskova et al. [51] demonstrated through standardized in vitro digestion that monocultures of *Lactobacillus acidophilus* and *Bifidobacterium breve*, as well as a commercial probiotic mixture containing nine bacterial cultures (*Bifidobacterium bifidum*, *Bifidobacterium breve*, *Bifidobacterium longum*, *Lactobacillus acidophilus*, *Lacticaseibacillus casei*, *Lactiplantibacillus plantarum*, *Lacticaseibacillus rhamnosus*, *Lactobacillus lactis* and *Streptococcus thermophilus*) at a daily dose of 9 × 10^9^ CFU, exhibited significantly higher viability when co-digested with complex non-dairy solid foods (e.g., meals containing meat and fiber) than when digested in simple matrices or beverages. Furthermore, viability increased when prebiotics were added.

Similar results were obtained by Treven et al. [92], who assessed the survival of commercial probiotics containing different species of *Lactobacillus* and *Bifidobacterium* during simulated gastrointestinal digestion in the presence of various non-dairy matrices. The authors observed that co-ingestion with a complex solid matrix such as porridge significantly improved bacterial survival compared to scenarios where the probiotic cells were consumed with water or juice, reinforcing the idea that the combination of complex carbohydrates and proteins helps to buffer acid and bile stress during gastrointestinal transit.

In this context, recent studies have highlighted clear differences even among non-dairy matrices, emphasizing the importance of a food’s physical structure. Wang et al. [93] demonstrated that durum wheat pasta, characterized by a dense, continuous matrix with high buffering capacity, protected *Lacticaseibacillus rhamnosus* GG much more effectively during simulated gastrointestinal digestion than a soy-based plant drink, despite both providing nutrients. The authors attribute this greater protection to carbohydrate and protein content and the structural architecture of solid food, which limits the diffusion of protons and digestive enzymes towards the probiotic cells. These results demonstrate that the structural and physicochemical complexity of the food matrix, as well as its nutritional composition, is decisive for the survival of probiotics during digestion.

The documented negative effects of processing, storage and gastrointestinal digestion emphasize the difference between the high levels of probiotics initially present in food and the lower levels that reach the intestine. This highlights the need for strategies to enhance survival. It is necessary to reduce technological stress during processing, provide structural protection, and strengthen microbial robustness through adaptive responses. These principles guide efforts to optimize processing conditions, apply encapsulation techniques, and develop stress adaptation or strain evolution approaches.

## 4. Strategies to Enhance Probiotic Survival: Protection and Adaptation

### 4.1. Resistance to Processing: Control of Parameters and Emerging Processing Technologies

As stated in the previous section, conventional food processing often exposes probiotics to stressful conditions, such as high temperatures, prolonged oxygen exposure, severe heat treatments, abrupt pH shifts, or extended processing times that can drastically reduce microbial viability and compromise the functional benefits of the final product. To mitigate these losses, two complementary approaches are essential: (i) strict control of key environmental parameters, including pH, oxygen and redox levels, fermentation dynamics, and substrate composition; and (ii) the use of emerging processing technologies which minimize cellular stress during product manufacture.

Controlling pH, oxygen and redox conditions, fermentation parameters (such as temperature and time), and the composition of the growth medium helps minimize probiotic stress, whether from acidification or oxidative damage, and enhances their survival in non-dairy products. Among these factors, pH is particularly critical. In a study on the fermentation of cereal-based probiotic beverages, the pH declined from 6.0–6.3 to 3.3–3.7 within 24 h, with the sharpest drop in the first six hours. *Limosilactobacillus reuteri* grew rapidly while the pH remained within its optimal range (4.5–6.8) during the first 6–12 h, reaching 11.70–12.96 log CFU/mL before further acidification halted growth; therefore, differences in cereal buffering capacity explained the higher counts observed in coix seed [94]. Oxygen and redox conditions represent another key determinant of probiotic stability. Although these parameters have been less studied in non-dairy systems, evidence from fruit-based matrices shows that oxygen exposure strongly affects survival during storage. In clarified apple juice containing *Lacticaseibacillus paracasei* ssp. *paracasei*, viability declined more rapidly when stored in PET plastic due to higher oxygen permeability, whereas glass packaging, allowing minimal oxygen ingress, preserved substantially higher counts. This pattern reflects the well-known sensitivity of probiotics to oxidative stress, linked to their lack of catalase enzyme and an electron transport chain [95].

Further evidence of the importance of controlling environmental parameters comes from work on oat-based non-dairy substrates. In an oat beverage fermented with nine lactic acid bacteria, incubation temperature and fermentation time had a marked influence on microbial performance and product functionality. Fermentations carried out at 30 °C generally resulted in higher viable counts (up to 3.2 × 10^8^ CFU/mL) and more desirable flavor development than those at 37 °C, while extended fermentation (24–72 h) and lower temperatures (25–30 °C) enhanced exopolysaccharide formation and viscosity when using *Lactobacillus delbrueckii* subsp. *bulgaricus* NCFB 2772. These effects were further amplified when the oat medium was supplemented with glucose, underscoring how temperature, time and substrate composition interact to shape fermentation dynamics and final product quality in non-dairy matrices [96].

Emerging processing technologies encompass a range of innovative, non-conventional methods, primarily non-thermal or minimally thermal, that aim to preserve microbial viability and product quality while reducing the intensity of conventional processing. Several of these techniques have been investigated for their ability to enhance probiotic survival in non-dairy matrices.

High-pressure homogenization (HPH) offers simultaneous homogenization, microbial inactivation and emulsion stabilization, while improving the nutritional quality of liquid foods during shelf life. Pressures below 200 MPa are classified as HPH, whereas higher levels fall within ultra-high-pressure homogenization (UHPH), reaching up to 400 MPa [97,98]. In mandarin juice containing *Ligilactobacillus salivarius* CECT 4063, HPH markedly influenced probiotic survival during fermentation and refrigerated storage (4 °C). Treatment at 20 MPa yielded the best results, with viability increasing to 8.58 log CFU/mL on day 2 and remaining above 8.45 log CFU/mL until day 7. Non-homogenized samples, in contrast, declined from 8.10 (day 1) to 5.20 log CFU/mL (day 10), while 100 MPa caused an early rise to 8.60 log CFU/mL (day 2) followed by a sharper decrease to 5.80 log CFU/mL (day 10). Therefore, the authors concluded that moderate HPH pressures enhance *L. salivarius* growth and stability of viable counts in this non-dairy matrix [99].

High-pressure processing (HPP) is a non-thermal technology that inactivates enzymes and pathogens using pressures between 100 and 800 MPa, thereby extending the shelf life of a wide range of foods, including plant-based and non-dairy products [100]. Recent studies indicate that this gentle preservation method can also stabilize plant-based fermented systems while maintaining probiotic viability. In sweet corn milk yogurt, HPP was applied after fermentation with *Lacticaseibacillus casei* 01. The untreated control reached 6.85 × 10^10^ CFU/g, while mild pressurization at 100 MPa preserved similarly high counts at 5.37 × 10^10^ CFU/g. In contrast, pressures ≥ 200 MPa markedly reduced viability, indicating that only gentle HPP conditions are suitable for protecting live probiotic cultures [101].

Sonication, a homogenization technique based on cavitation at frequencies above 20 kHz, is used in plant-based beverages to reduce particle size, improve protein solubility and enhance stability by limiting sedimentation, while also helping to inactivate undesirable microorganisms [100]. In cantaloupe melon juice, ultrasonic pre-treatment acted as an effective emerging technique to support probiotic viability, as it eliminated the lag phase, increased sugar availability and enabled *Lacticaseibacillus casei* B-442 to reach 9 log CFU/mL after 8 h of fermentation, significantly outperforming the non-sonicated control. This enhanced performance was sustained during refrigerated storage (42 days), with the product maintaining viabilities above 8.8 log CFU/mL [102].

### 4.2. Structural Protection Strategies: Encapsulation Techniques

Microencapsulation is a technological process in which probiotic microorganisms are enclosed within a microscopic capsule that acts as protective barrier. In food applications, this technique has been widely employed to improve stability during processing and storage, preventing premature loss of viability and therefore ensuring that effective levels of microorganisms are retained within the ranges recommended for health benefits [103,104]. Beyond technological benefits, microencapsulation also plays a critical role in gastrointestinal survival. By creating a physical barrier, encapsulated cells are shielded from extreme conditions such as gastric acidity, bile salts and digestive enzymes, which would otherwise cause substantial reductions in viability. This protection allows more viable cells to reach the small intestine and colon, where they can exert their beneficial effects on the host [70,105].

Recent reviews have synthesized the advances achieved in probiotic microencapsulation over the last years. Sbehat et al. [106] highlighted its application across diverse food matrices, emphasizing protection against harmful conditions and the possibility of combining probiotics with other functional ingredients such as polyphenols, prebiotics or omega-3 fatty acids. In parallel, Vivek et al. [107] provided a comprehensive overview of the most widely used techniques, carrier materials and current trends, particularly in the development of non-dairy probiotic fruit juice powders. Together, these reviews underline both the technological progress and the expanding scope of microencapsulation strategies in functional food design. However, despite their demonstrated effectiveness at laboratory scale, encapsulation systems still face relevant challenges for industrial implementation. As reported by Burgain et al. [103], the incorporation of microencapsulation requires additional processing steps that complicate food manufacture and increase production costs, mainly due to the need for controlled processing conditions and specialized materials and processing technologies.

The most commonly used techniques for probiotic microencapsulation today include extrusion (external ionic gelation), emulsion with internal gelation, spray-drying, freeze-drying (lyophilization), spray chilling, fluidized bed coating/drying, multilayer (layer-by-layer) methods, and simple or complex coacervation [108]. Each technique presents specific advantages and limitations in terms of viability preservation, process complexity and scalability. In this context, some methods are particularly suitable for industrial scale-up and cost reduction (e.g., spray-drying and spray-chilling), whereas others, although highly protective and allowing very high cellular viability by avoiding extreme temperatures (e.g., extrusion, coacervation, freeze-drying), are more restricted by processing constraints and scalability issues [109]. Moreover, the performance of these systems strongly depends on the selection of carrier materials, which must be adapted to the probiotic strain, the target food matrix, and the processing and storage conditions. Polysaccharides such as alginate, pectin, gum arabic, and starches, as well as proteins (whey, casein, zein, soy) and lipids, are commonly used alone or in blended systems to achieve adequate protection, delay release until the intestine, and ensure microbial survival during storage and gastrointestinal transit [110].

According to recent studies on specific probiotic strains, microencapsulation has consistently been shown to significantly enhance the resistance of microorganisms to gastrointestinal stress. Qi et al. [111] demonstrated that strains such as *Saccharomyces boulardii* and *Enterococcus faecium*, when encapsulated using emulsion and internal gelation techniques, exhibited higher survival rates under adverse conditions of high temperature, high humidity, and simulated gastric and intestinal environments, compared to non-encapsulated cells. These findings are supported by the data presented in Table 2, which summarizes recent studies on the improvement of probiotic viability through microencapsulation.

The primary drawback frequently reported in the literature regarding microencapsulation in non-dairy foods is the occurrence of sensory changes that may affect consumer acceptance. Among the most commonly cited effects are alterations in texture, variations in viscosity, the perception of discernible particles in the mouth, and, in some cases, changes in flavor, aroma, or appearance [112]. In a study on yoghurt, it was also observed that, although microencapsulation with whey protein isolate (WPI) or alginate better maintained probiotic viability during storage and under simulated gastric juice conditions, there were differences in viscosity, syneresis, and minor changes in acidity compared with yoghurt containing free probiotics. These changes may affect the perception of texture and flavor, although not necessarily in a negative way [113].

Nanoencapsulation is an advanced technique derived from nanotechnology that involves incorporating bioactive compounds into nanometer-sized structures, typically less than 100 nanometers. This technology enables controlled and targeted release of active ingredients, enhancing their stability, bioavailability, and protection against adverse conditions in the gastrointestinal environment [114]. In the context of non-dairy foods, nanoencapsulation offers significant advantages, such as improved solubility and stability of nutrients, antioxidants, and probiotics. Additionally, it facilitates the specific release of these compounds at the appropriate site in the digestive tract, optimizing their absorption and efficacy [115].

There are various nanoencapsulation techniques that are prominent in the food industry due to their ability to enhance stability, bioavailability, and controlled release of bioactive compounds. Emulsification is a widely employed technique for encapsulating lipophilic compounds [116]. Coacervation involves liquid–liquid phase separation to form a polymer-rich phase that encapsulates the compound of interest [117]. Solvent evaporation consists of dissolving the bioactive compound along with a polymer in an organic solvent, followed by the evaporation of the solvent to form a solid matrix that encapsulates the active ingredient [118]. Supercritical fluid technology, such as supercritical carbon dioxide, is used for nanoparticle formation without the need for organic solvents. These techniques offer various advantages in improving stability, bioavailability, and controlled release of bioactive compounds in non-dairy foods [119].

Although nanoencapsulation offers important advantages, including enhanced stability, targeted delivery, controlled release, and improved protection of probiotics against environmental and gastrointestinal stresses, it is not always superior to microencapsulation for food applications. Despite its promising performance, nanoencapsulation still faces important limitations, including aggregation, regulatory uncertainties, oxidation susceptibility, and high manufacturing costs. In contrast, microencapsulation continues to dominate food applications because of its robustness, lower production costs, established encapsulation technologies, and recognized regulatory status [120,121].

**Table 2:** Encapsulation techniques for probiotic survival within non-dairy matrices under processing, storage and gastrointestinal digestion.

Non-Dairy Matrix/Food	Probiotic Strain(s)	Encapsulation Technique	Processing/Storage Conditions	In Vitro Digestion Conditions	Viability Results	Reference
Chocolate	*Lactobacillus acidophilus* (La-5), *Lacticaseibacillus rhamnosus* (LGG), *Fructilactobacillus sanfranciscensis*, *Lactiplantibacillus plantarum*, *Lacticaseibacillus casei* 431^®^, *Bifidobacterium animalis* subsp. *lactis* (BB-12), and *Streptococcus thermophilus*.	Encapsulation via freeze-drying using two emulsion-based formulations: (i) Cocoa powder: Na-alginate at 10:1 ratio, and (ii) cocoa powder: Na-alginate: fructooligosaccharides at 10:1:2 ratio.	Thermal processing: chocolate with EP and non-EP heated at 40, 50, and 60 °C. Storage: EP and non-EP chocolates at 4 °C and 25 °C for 90 days.	GI digestion and colonic fermentation (EP and non-EP chocolates): digestion at 0, 2, 4 h. Fermentation with fecal inoculum (healthy 32-year-old male) at 1, 24, 48, 72 h.	Encapsulation efficiency: *L. casei* (93.40%, formulation i) and *L. acidophilus* La5 (95.36%, formulation ii). After thermal exposure (60 °C) in chocolate, EP strains retained > 9 log CFU/g vs. ~4 log in non-EP. During storage, EP remained ≥ 7 log CFU/g (therapeutic level), whereas non-EP declined. In GI digestion, EP reached ~8 log CFU/g and increased to 10.50 log CFU/g during colonic fermentation.	[122]
Dried apple slices	*Ligilactobacillus salivarius* subsp. *salivarius*	Encapsulation via high-pressure homogenization (70 MPa) using sodium alginate–Tween 80–sunflower oil emulsion; rupture with CaCl_2_ and recover by centrifugation.	Vacuum impregnation: mandarin juice with EP and non-EP, 50 mbar (10 min) + atmospheric pressure (10 min). Air-drying: 40 °C (24 h). Storage: opaque sealed bags at room temperature, 30 days.	GI digestion (EP and non-EP apples): pepsin 0.6% (pH 3, 37 °C, 90 min) → bile 10% (pH 8) → bile 0.3% + pancreatin 0.1% (37 °C, 90 min); applied to samples stored at 0, 7, 14, 21, and 30 days.	Initial counts in mandarin juice: EP (8.18 log CFU/g) and non-EP (8.37 log CFU/g). After apple impregnation: EP (7.23 log CFU/g), non-EP (7.34 log CFU/g). During air-drying: EP retained 7.19 log CFU/g, non-EP dropped to 6.71 log CFU/g. After 30 days of storage: EP survival 39%, non-EP 19%. EP also showed greater tolerance during GI digestion, especially under low pH and bile stress.	[123]
Radish kimchi	LAB KCC-42 (endophytic lactic acid bacterium closely related to *Lactiplantibacillus plantarum*)	Encapsulation via freeze-drying, using alginate–chitosan beads gelled in CaCl_2_/HEPES.	Storage: EP and non-EP radish kimchi at 27 °C for 0, 2, 4, 8, 12, and 24 days under varying pH conditions.	GI digestion (EP and non-EP only): SGF (pepsin, pH 1.9, 37 °C, 2 h) → SIF (bile 3.6% and pancreatin 0.1%, pH 6.5, 37 °C, 4 h). Samples collected at 30, 60, 90, and 120 min.	EP (LAB KCC-42) demonstrated enhanced viability compared to non-EP in kimchi fermented at 27 °C for 3 weeks, showing greater resistance under acidic conditions (pH 3.8). EP showed greater viability (7.48 × 10^5^ log CFU/mL) and tolerance to acidic/pancreatin conditions than non-EP (6.50 × 10^5^ log CFU/mL).	[124]
Mimic beverage systems	*Lactiplantibacillus plantarum* JYLP-326	Encapsulation via extrusion, using sodium alginate or sodium alginate-gelatine hydrogels (2% *w*/*v*, 3:1); sprayed into CaCl_2_ (0.1 M); hardened and rinsed with saline.	Heat treatment: only EP and non-EP, at 37–60 °C for 5 min. Storage: only EP and non-EP, refrigerated at 4 °C for 6 days.	GI digestion (EP and non-EP only): SGF (pepsin 0.3%, pH 2.0, 37 °C, 40 min); viability assessed at 0–40 min. SIF (trypsin 1%, bile salts 0.1%, pH 7.0, 37 °C, 4 h); release monitored hourly.	Encapsulation efficiency was >97% for EP. Under heat treatment, non-EP lost 5.7 log CFU, while EP lost only 0.2 log CFU. During refrigerated storage, non-EP lost > 2.1 log CFU, whereas EP lost only 1.4 log CFU. During GI digestion, EP showed 97.8% survival in SGF, while non-EP viability dropped. In SIF, EP released > 9.0 log CFU; non-EP showed poor survival and limited release.	[125]
Mango juice	*Lactiplantibacillus plantarum*	Microencapsulation via internal gelation using SA–SPI (sodium alginate–soy protein isolate) emulsions (2:0 to 2:6), germ-free oil, and Teen-80; rupture with CaCl_2_ and rinse with saline solution.	Heat treatment of EP only: 63–72 °C (5 min). Storage of EP only: stored at 4 °C (28 days). Storage in mango juice: pasteurized juice inoculated with EP; incubated (37 °C, 12 h), stored at 4 °C (28 days).	GI digestion (EP only): SGF (pepsin 1000 U/mL, pH 3, 2 h) → SIF (bile salts 0.3%, pH 6.8, 3 h) under anaerobic conditions; performed before EP incorporation into mango juice.	SA:SPI 2:4 showed highest encapsulation efficiency (95.92%) and best overall probiotic protection. It maintained superior viability under refrigeration storage (7.69 log CFU/g), in simulated gastric and intestinal fluids (8.31 and 8.62 log CFU/mL), and during mango juice storage (only 0.94 log CFU/mL loss vs. 3.4 in non-EP). SA:SPI 2:2 showed best heat resistance (9.70 log CFU/mL at 63 °C).	[126]
Green soybean yogurt	*Bifidobacterium breve* TISTR 2130	Microencapsulation via external gelation in sodium alginate (1.5–2.5%) with calcium lactate (1–2%) in green soy milk.	Storage: Yogurt prepared by fermentation, followed by addition of EP and stored at 4 °C for 0, 0, 5, 10, 15 and 20 days.	GI digestion (EP and non-EP only): SGF (pepsin 0.03%, pH 2, 37 °C, 2 h) → SIF (bile 3.6% and pancreatin 0.8%, pH 7.4, 37 °C, 3 h).	Encapsulation efficiency of 99.8% with alginate and calcium lactate both at 2%. EP survived gastrointestinal digestion with >6 log CFU/mL; non-EP undetectable after 2 h. During storage, EP maintained viability > 6 log CFU/mL after 10 days.	[127]
Sour cherry juice	*Enterococcus faecium*	Microencapsulation via extrusion, using 2% sodium alginate; dropped into CaCl_2_ (4% *w*/*v*); hardened and rinsed with DI water.	Heat tolerance: only EP and non-EP, at 60–80 °C for 0–10 min. Acid tolerance: only EP and non-EP, at pH 1.5–2.5 (90 min; fresh and 30-days in sour cherry juice).	GI digestion (non-EP, EP fresh and 30-days in juice): SGF (pepsin 0.3%, pH 2.0, 37 °C, 1–2 h) → SIF (pancreatin 0.2%, bile salts 0.6%, pH 7.0, 37 °C, 1–2 h).	Encapsulation increased resistance to heat, acid, and gastrointestinal conditions, with higher D-values reflecting thermal resistance (9.41–1.26 min for EP vs. 4.87–0.43 min for non-EP), lower acid-induced reductions (1.31–2.37 log CFU/mL for EP fresh vs. up to 4.18 log CFU/mL for non-EP), and improved GI survival (reductions of 2.97 log CFU/mL for EP fresh vs. up to 4.77 log CFU/mL for non-EP). EP 30-days showed moderate decline.	[128]
No final food matrix	*Lactobacillus acidophilus* (LA5), *Lacticaseibacillus rhamnosus* 23527 LGG, *Bifidobacterium bifidum* and *Bifidobacterium animalis*	Nanoencapsulation via electrospinning using corn starch (CS), sodium alginate (SA), and CS/SA/probiotic (CSP, 10 log CFU/mL) at a 5:1:2 *v*/*v*/*v* ratio; optimized at 1.5 mL/h, 24 kV, 12 cm; freeze-dried post-collection.	Low pH: EP and non-EP stability at pH 2.5, 4.5, and 7.0 for 6 h at 37 °C.	GI digestion (EP and non-EP): SGF (pepsin 0.3%, pH 2.5–3.0, 37 °C, 2 h); viability at 0–120 min. SIF (trypsin 2%, bile salts 0.2%, pH 6.8, 37 °C, 4 h); viability at 0–240 min.	EP improved acid resistance; lactobacilli retained 84.5% viability at pH 4.5 (4 h) and 100% at pH 2.5, while bifidobacteria dropped to 42.1% at pH 4.5 and showed no survival at pH 2.5. EP improved survival in GI digestion; viability at 120 min reached 90% (lactobacilli) and 84.1% (bifidobacteria), and at 240 min 54.8% and 63.1%, respectively.	[129]
No final food matrix	*Lactococcus lactis* ATCC 11454, *Lactiplantibacillus plantarum* ATCC 14917, *Lacticaseibacillus paracasei* ATCC 334, and *Saccharomyces cerevisiae* ATCC 204508/S288c	Nanoencapsulation via ionic gelation of synbiotic (pomegranate peel + probiotics, 10^12^ CFU/mL) with sodium alginate (3%, *w*/*v*) and Tween-80 (0.5%); dropped into CaCl_2_ (2.22 mol/L); centrifuged; stored at −80 °C.	Storage: 6 months at room temperature (25 °C) for encapsulated and non-encapsulated synbiotics.	Gastric digestion (encapsulated and non-encapsulated synbiotics): SGF (porcine pepsin, pH 3.0, 37 °C, 2 h).	Nanoencapsulation enhanced both storage and gastric tolerance of synbiotics. After storage, viability reached 5.48 log CFU/g for encapsulated and 4.74 log CFU/g for non-encapsulated forms. Under gastric conditions, viability was 5.65 log CFU/g and 4.97 log CFU/g, respectively.	[130]

EP: Encapsulated Probiotics; SGF: Simulated Gastric Fluid; SIF: Simulated Intestinal Fluid.

As mentioned earlier in the microencapsulation section, evidence also shows that nanoencapsulation can affect sensory properties of foods. Ju et al. [131] conducted a study with soy milk developed using nanotechnology, comparing commercial samples with laboratory-formulated ones. They evaluated attributes such as appearance, odor, flavor, mouthfeel (texture), and presence of particles. Consumers rated negatively those samples showing visible particles or a rough mouthfeel, residual raw soy flavor, or a greyish color, relative to samples with a more uniform texture and smoother attributes. In another study using α-lactalbumin nanotubes to encapsulate soy isoflavones, it was found that, besides improving the compound’s functional stability and release under simulated digestion, there were changes in the rheological properties of the soy milk—an increase in viscosity and perceptible changes in texture [132]. These findings suggest that while nanoencapsulation offers functional advantages (greater protection, controlled release, etc.), possible effects on flavor, aroma, texture, and appearance need careful consideration to ensure final product acceptability.

### 4.3. Stress Adaptation and Strain Evolution Strategies

Stress adaptation in probiotic strains refers to the controlled exposure to suboptimal conditions, such as moderate heat or cold, desiccation, an acidic pH, bile fluctuations, oxidative challenges, or osmotic variations, during cultivation or prior to final formulation. This controlled exposure to suboptimal conditions activates cellular defense mechanisms, including the production of protective proteins (e.g., heat shock and cold shock proteins), the accumulation of compatible solutes, and structural modifications of cell membranes. Collectively, these responses enhance microbial resilience and facilitate the subsequent survival and stability of cells into diverse non-dairy food matrices, where they must withstand both processing stresses and gastrointestinal transit [90,133].

Numerous studies have demonstrated that adapted microbial cells can develop cross-tolerance. For instance, cells exposed to mild stress conditions may increase their resistance either to the same stressor or to different, non-exposed stressors, including high temperature, acidity, or the presence of oxygen [134,135,136,137]. Such adaptive responses have been exploited to evaluate the resilience of common probiotic strains under challenging conditions. For instance, *Lacticaseibacillus casei*, *Lactobacillus acidophilus* and *Lactiplantibacillus plantarum* subsp. *plantarum* have been subjected to acidic pH (4–6.5), salt (1–7%) and sucrose (0.1–0.7 M), and pre-adaptation markedly enhanced their stress tolerance. *L. acidophilus*, unable to grow above 1% NaCl, grew up to 7% after pre-adaptation; moreover, under salt stress, its lag phase decreased from 23.57 h to 3.91–7.08 h, and maximum growth rates increased up to 10-fold under sucrose stress. *L. casei* showed similarly shorter lag phases (26.86 h to 6.49–7.86 h at 5% NaCl) and 2–3-fold faster growth under sucrose. *L. plantarum*, already more tolerant, further reduced its lag phase (32.01 h to 11.59 h at 7% NaCl) and showed moderate increases in growth rate across salt, sucrose and acidic stresses [83]. In other cases, lactic acid bacteria isolated from traditional fermented foods, for example *Lactobacillus kefiranofaciens* subsp. *kefiranofaciens* M1 from Taiwanese kefir grains, were exposed to heat, cold, acid and bile salts, resulting in improved stress tolerance [138].

When probiotic cells are exposed to sublethal stress, morphological changes can be observed. Alterations such as elongated shapes or increased cell size have been reported under starvation or osmotic stress in *Lactobacillus* and *Lacticaseibacillus casei* [139,140]. Superficial modifications of *Ligilactobacillus salivarius* cells led to increased hydrophobicity following sublethal homogenization pressures or trehalose addition in mandarin juice, and alterations in the growth kinetics of probiotic microorganisms were also observed after exposure to stressful condition [99]. A correlation between cell morphology and strain stability during key processing steps, including extrusion, lyophilization, freezing, dehydration and storage, has been highlighted [140,141]. The cellular response to suboptimal growth conditions was shown to be strain-dependent and varied according to the type of stress applied. Consequently, screening for the most appropriate measurement parameters and defining the limiting values for each strain are essential.

To identify and understand the adaptive mechanisms employed by probiotic cells, as well as to evaluate their subsequent metabolite production, the analysis of gene and protein expression under stressful conditions represents a valuable approach. Jung & Lee [142] examined the gene expression of *Lactiplantibacillus plantarum* under acidic conditions. The differentially expressed genes indicated that transport functions were affected, and acidity during fermentation was shown to regulate intracellular leucine transport. Proteomic analysis conducted in *Lactobacillus kefiranofaciens* subsp. *kefiranofaciens* M1 subjected to different sublethal stresses revealed that 27 proteins were differentially expressed between adapted and non-adapted cells [138]. Similar findings are summarized in Table 3, where several adapted probiotic strains demonstrate measurable improvements in stress tolerance relative to their parental strains.

Directed evolution is a process based on Darwin’s theory of natural selection. It involves adaptation to highly stressful environments through spontaneous DNA mutations. Organisms acquiring advantageous mutations thrive, reproduce, and become dominant under the specific stress. The first step is the generation of a library of genetic variants [143]. Those closest to the desired traits are selected for breeding, after which random mutagenesis or recombination is applied to create new genotypic variants from this pool [143]. This two-step cycle is repeated as many times as required. In recent years, biotechnological techniques have been refined to identify the genetic codes of acquired mutations and to activate, delete, or modify the responsible genes, thereby increasing throughput and reducing human intervention [144,145]. However, the fundamental evolutionary process remains unchanged.

Through such advances, directed evolution has been applied to generate industrially relevant phenotypes, including enhanced stress resistance (e.g., acid or ethanol tolerance), increased production of compounds of interest such as ferulic acid and flavonoids, and food products with tailored sensory properties, particularly in wine and beer. As shown in Table 3, strains adapted through this approach exhibit measurable improvements in stress tolerance, viability, and functional stability, confirming its value for the development of more robust probiotic products.

Despite these promising applications, it is important to acknowledge the limitations of directed evolution. Standard protocols are based on iterative rounds of mutagenesis and selection, typically requiring several cycles of diversity generation and screening to obtain improved variants, a workflow that is inherently resource-demanding, labor-intensive and time-consuming [146,147]. Furthermore, adaptive mutations frequently entail fitness costs, as improvements in performance under selective conditions may reduce fitness in alternative environments and lead to pleiotropic side-effects that alter other phenotypic traits [148,149,150]. Beyond these biological and operational constraints, approaches based on mutagenesis or gene editing also face significant regulatory and societal barriers. Following the ruling of the Court of Justice of the European Union, such techniques fall under the European Union Genetically Modified Organism regulatory framework, which imposes substantial restrictions on their use [151]. At the same time, public perception of biotechnology is shaped by perceived risk and limited understanding, meaning that even clearly beneficial innovations are often approached with caution, resulting in a general reluctance to accept genetically modified foods [152].

Beyond the technological and biological considerations of directed evolution, regulatory frameworks impose important constraints on the use of mutagenesis-based or gene-editing approaches in food-grade probiotics. In the United States, probiotic microorganisms used in foods are regulated under the U.S. Food and Drug Administration (FDA) through the Generally Recognized as Safe (GRAS) framework, which permits marketing when qualified experts determine—through scientific procedures or common use in food—that the substance is safe under its intended conditions of use [153]. GRAS conclusions rely on publicly available evidence and must include information on identity, manufacturing, exposure, and the scientific basis for safety [153]. This framework does not address mutagenesis, directed evolution, or other strain-level genetic modifications. However, in the clinical context, Live Biotherapeutic Products (LBPs) require an Investigational New Drug (IND) application, in which sponsors must provide detailed descriptions of the strain’s biological name, culture history, phenotype, genotype, and any intentional genetic modifications [154]. In the European Union, the Qualified Presumption of Safety (QPS) system requires that microorganisms intentionally added to food or feed be evaluated for taxonomic identity, body of knowledge, and safety concerns, and specifies that strains must not harbor acquired antimicrobial resistance genes; these qualifications also apply to genetically modified strains, as the QPS approach can only be extended when the modification does not give rise to safety concerns and when any gene of concern has been removed [155]. From an evolutionary perspective, recent analyses highlight that probiotics and LBPs can undergo rapid genetic and population-level adaptation within the gastrointestinal tract, driven by mutation, selection pressures, and horizontal gene transfer, underscoring the importance of considering in vivo evolutionary dynamics when evaluating regulatory compliance and safety of strains developed through directed evolution [156].

The long-term stability of stress-adapted phenotypes should also be considered when evaluating the industrial applicability of biological adaptation strategies. The persistence of enhanced stress resistance following the removal of selective pressure depends largely on the underlying mechanisms involved. If adaptation results from stable genetic changes, such as mutations that accumulate during adaptive laboratory evolution, the acquired phenotype is generally maintained over multiple generations and during storage, unless the mutations impose a significant fitness cost. In contrast, adaptations based on physiological conditioning, transcriptional reprogramming or epigenetic regulation may be transient and progressively disappear once cells are no longer exposed to the inducing stress. Recent studies have shown that bacterial stress responses can involve epigenetic mechanisms, such as DNA methylation patterns, which temporarily modulate gene expression without altering the genome sequence. While these mechanisms may confer enhanced tolerance during processing and short-term storage, their stability during prolonged storage and subsequent propagation is less predictable. Therefore, when developing stress-adapted probiotic cultures, evaluation of both genetic and epigenetic stability should be included, as the long-term maintenance of the desired phenotype is essential for industrial implementation and product consistency [157,158].

**Table 3:** Stress adaptation and evolution strategies to enhance probiotic survival and functionality.

Strategy	Strain	Conditions of Adaptation/Evolution	Challenge Conditions	Results	Comments	Reference
Stress adaptation	*Lactiplantibacillus plantarum* KLDS 1.0628	Heat (45 °C, 1 h), cold (15 °C, 1 h), oxidative (1 mmol/L H_2_O_2_, 1 h, 37 °C), acid (pH 4.0, 1 h, 37 °C), bile salts (0.2%, 1 h, 37 °C), osmotic (2% NaCl, 1 h, 37 °C). Control: non-adapted cells in MRS, 37 °C, 1 h. Both groups exposed to 60 °C, 1 h.	Heat (60 °C, 1 h), cold (−20 °C, 14 d), oxidative (5 mmol/L H_2_O_2_, 1 h, 37 °C), acid (pH 2.5, 1 h, 37 °C), bile salts (0.5%, 1 d, 37 °C), and osmotic stresses (8% NaCl, 1 d, 37 °C).	Control (non-adapted): 0.08% survival. Heat adaptation was most effective against thermal stress (2.51%). Acid adaptation improved tolerance to cold (1.18%), oxidative (0.51%) and acid stress (0.10%). With bile salts, specific adaptation gave highest survival (2.35%), followed by acid and NaCl. Against osmotic stress, 2% NaCl adaptation yielded best result (2.19%).	Changes in cell-membrane fatty-acid composition. The adaptations altered the proportion of saturated versus unsaturated fatty acids in the cell membrane. A notable increase in cyclopropane fatty acid was observed in the adapted cells, which suggests that *L. plantarum* adjusts the fluidity, elasticity and stiffness of its membrane to withstand stressful conditions.	[134]
Stress adaptation	*Lactobacillus kefiranofaciens* M1	Heat (37 °C, 1 h), cold (20 °C, 1 h), acid (pH 5.0, 1 h, 30 °C), and bile salts (0.05%, 1 h, 30 °C). Control: 30 °C for 1 h.	Heat (52 °C, 2 h), cold (−20 °C, 12 d), acid (pH 3.0, 6 h, 30 °C), and bile salts (0.2%, 2 h, 30 °C).	Thermal tolerance (52 °C) increased 53.5-fold with heat, acid and bile salts adaptation. Cold tolerance (−20 °C, 12 d) rose up to 9.60-fold after acid, bile salts and cold adaptation. Acid tolerance (pH 3.0) improved up to 9.92-fold with acid and bile salts adaptation. Resistance to bile salts (0.2%) increased up to 59.30-fold with all adaptations.	Twenty-seven proteins showed altered expression after adaptation to heat, cold, acid and bile salts, influenced by multiple pretreatments. They were involved in carbohydrate metabolism, pH homeostasis, stress response, transcription, translation, and amino acid and nucleotide metabolism.	[138]
Stress adaptation	*Fructilactobacillus sanfranciscensis* CB1	Cells were acid-adapted for 1 h at pH 5.0, 30 °C with lactic acid. Control and mutant cells (CB1-5R, CB1-7R) remained at pH 6.4 under identical conditions. Chloramphenicol inhibited protein synthesis.	Cells were challenged in acidic environments (pH 3.2–4.0, 10 h, 30 °C) using lactic acid, HCl, or lactic–acetic acid mixtures. Cold (10 °C) and osmotic (5% NaCl) stress tested for 24 h in MRS medium.	pH 3.4–3.2 reduced viability; adaptation at pH 5.0 for 1 h increased tolerance (4 × 10^3^), blocked by chloramphenicol. Mutants CB1-5R and CB1-7R showed similar tolerance. Adapted cells and mutants grew better at 10 °C and 5% NaCl (8.6 log CFU/mL) vs. 7.0 in non-adapted cells, despite initial loss.	The mutants CB1-5R and CB1-7R showed protein changes versus non-adapted cells, with similar profiles but distinct from adapted cells. Heat-shock proteins identified included DnaJ, DnaK, GroES and GrpE; only GrpE increased in both mutants and adapted cells.	[159]
Stress adaptation	*Bifidobacterium longum* JDM301	JDM301AR, an acid-resistant strain, was isolated after 150 anaerobic subcultures of JDM301 in modified MRS at pH 6.5 and 37 °C	Acid (pH 3.5), oxidative (1.25 mM H_2_O_2_), and osmotic (0.5 M NaCl) stress tested after 1.5 h anaerobic incubation at 37 °C.	JDM301AR showed enhanced tolerance to acid, oxidative, and osmotic stress, with survival rates around 60%, 60%, and 40% respectively, compared to approximately 40%, 40%, and 20% in the parental strain.	The change in the fatty-acid composition of the cell membrane (an increase in C14:0) in the adapted strain JDM301AR suggests that membrane modifications are key to acid-stress tolerance.	[160]
Stress adaptation	*Lacticaseibacillus rhamnosus* hsryfm 1301	Oxidative (0.5–2 mM H_2_O_2_, 1 h), heat (4–50 °C, 1 h) and osmotic (0.15–0.6 M NaCl, 1 h) stresses. Control: static cultures.	Oxidative (5 mM H_2_O_2_, 1 h), heat (37–60 °C, 1 h), and osmotic (0–1.0 M NaCl, 1 h) stresses.	Highly sensitive to 2–5 mM H_2_O_2_ (0.01% survival); 0.5 mM and 2 mM pretreatment raised survival to 66% and 100%. Heat shock at 46 °C increased oxidative tolerance 12-fold; 50 °C reduced survival by 90%. Osmotic (0.6 M NaCl) and acid (pH 2.5) shocks improved survival under 2 mM H_2_O_2_, not at 5 mM.	Oxidative pretreatment may enhance the resistance of *L. rhamnosus* hsryfm 1301 to heat and oxidative stress during air-drying and improve its oxidative stability during storage.	[161]
Directed evolution	*Saccharomyces cerevisiae* EC1118, T73, and IFI473	~140 generations in YPGal at 25 °C, 180 rpm; serial 10% transfers; increasing 2-deoxyglucose (2DG) (50–200 mg/L); evolved strains and clones used for rosé and white juice fermentations.	Strains were inoculated in jellified synthetic grape juice under stress conditions: high ethanol (6–10% *v*/*v*), low/high temperature (12 °C, 37 °C), oxidative stress (10 mM H_2_O_2_), and sulphite (120 mg/L K_2_S_2_O_5_).	Evolved populations reduced acetic acid (EC1118: 7.01 → 1.46 mg/g) and showed strain-specific glycerol increases (IFI473: 31.65 → 54.16 mg/g) during aerobic grape juice fermentation. Only EC1118 clones consistently lowered acetic acid (<2 mg/g), T73 and IFI473 showed no clear improvement. Promising EC1118 clones reduced acetic acid in shake flasks (EF1C: ~2.5 mg/g), but not in bioreactors.	Directed evolution of *S. cerevisiae* lowered acetic acid production under aerobic conditions while maintaining fermentative capacity. EC1118 clones (EF1A, EF1C) were selected as they combined stress tolerance with consistent acetic acid reduction.	[162]
Directed evolution	*Lactobacillus acidophilus* CGMCC 1.1878	DNA of *L. acidophilus* extracted to amplify *faeLac* (feruloyl esterase gene), cloned into pET28a, expressed in *E. coli* BL21 (DE3), and mutated by Quick-change PCR.	Not a stress/challenge condition; mutants were evaluated for Ferulic acid (FA) release from wheat bran using xylanase + Feruloyl esterase (FAE) in phosphate buffer (pH 7.0, 25 °C, 150 rpm, 24 h).	FAELac optimal at 25 °C and pH 7.0; >80% activity at pH 7–10; inhibited by Cu^2+^ and pyridoxal phosphate (PLP). Kinetic parameters: *K_m_* 3.57 mM, *k*_cat_/*K_m_* 321.8 M^−1^·S^−1^. Mutants Q134T and Q198A increased catalytic efficiency up to 5.4-fold; Q134T released 6.60 mg/g FA from WAX (2.24-fold).	Directed evolution improved FAE performance, and this Study highlights enzyme homogeneity and substrate-specific adaptability, supporting future FAE processing and economic FA production.	[163]
Adaptative evolution	*Lacticaseibacillus casei* 12A and ATCC 334	MRS acidified (pH < 6.5); strains 12A/ATCC 334 ± ΔmutS; 37 °C serial transfers; pH 5.5 → 4.0/100 d; samples every ~5 d + PCR. Streaked pH 4.0 MRS; anaerobic 37 °C/20 d; 7 colonies tested; best isolate chosen.	Growth at pH 4.0: stationary-phase cells in MRS pH 4.0 (96-well, 37 °C, 17 d); final pH and lactic acid measured. Acid resistance: early stationary-phase cells (~10^10^ CFU/mL) in MRS pH 3.0–2.0 (HCl, 1 h, 37 °C); survival by plating.	Adapted strains (12A-AE, 334-AE, 12A-MAE, 334-MAE) grew faster and to higher density at pH 4.0 than parental; all showed lower final pH and more lactic acid, with MAE strains producing the most. At pH 3.0 all survived, at pH 2.0 none; at pH 2.5 survival was >10-fold higher in 12A-MAE vs. parental, while 12A-AE showed none.	Adaptive evolution + mutS inactivation improved acid tolerance; mutations in NADH dehydrogenase (ndh), phosphate transport ATP-binding protein (pstB), and histidine protein kinase (hpk) further enhanced growth, lactic acid production, and survival at low pH.	[164]
Experimental evolution	*Saccharomyces cerevisiae* (haploid, diploid, tetraploid lines)	Turbidostat cultures (30 °C, pH 5.0, 250 rpm, aerobic) in ethanol-containing medium (yeast extract, bactopeptone, glucose, chloramphenicol, antifoam); ethanol increased stepwise 6 → 12% (*v*/*v*) over ~200 generations (~2 y).	Fitness assays in 9% ethanol; growth tests on plates up to 11% ethanol; competition haploid vs. diploid strains.	Adapted populations showed increased ethanol tolerance, growing up to 11% EtOH vs. ancestral strains. Haploid and tetraploid lines converged to diploidy, conferring fitness advantage at 9% EtOH. Mutator phenotypes (e.g., MSH2) and CNVs (e.g., chromosome III duplication) supported adaptation.	After turbidostat ethanol evolution, cells adapted via diploidization, clonal interference, mutator phenotypes and CNVs, with mutations affecting stress response, DNA repair and respiration. Key adaptive alleles (PRT1, VPS70, MEX67) were identified, highlighting industrial relevance.	[165]
Directed evolution	*Saccharomyces cerevisiae* C800 (expressing SmF3′H = flavonoid 3′-hydroxylase and SmCPR = cytochrome P450 reductase, both from *Silybum marianum*)	Promoter library (20 gradients) via yeast recombination; error-PCR mutagenesis of SmF3′H/SmCPR; high-throughput colorimetric screening in SD-Ura and YPD media with (2S)-naringenin.	Fermentation with precursor (2S)-naringenin (up to 5 g/L) in 5-L bioreactor (30 °C, 600 rpm, 3 vvm airflow, pH 5.5) using feeding medium with glucose, amino acids, vitamins and trace metals.	Production of (2S)-eriodictyol increased from ~58 mg/L in the parental C800 strain to ~3.3 g/L in the evolved variant (C800P05mut12) under 5-L bioreactor conditions (30 °C, pH 5.5, 600 rpm, 3 vvm), representing a ~57-fold improvement in production.	Promoter balancing and directed evolution of SmF3′H/SmCPR improved enzyme activity and pathway flux. High-throughput colorimetric screening enabled rapid selection. Demonstrates yeast as an efficient platform for flavonoid P450 expression with industrial relevance.	[166]

## 5. Conclusions

The incorporation of probiotics into non-dairy food matrices is a rapidly growing research area driven by the demand for functional plant-based products. However, ensuring adequate viability throughout processing, storage, and gastrointestinal transit remains a critical challenge due to multiple and often overlapping environmental stresses.

Overall, probiotic performance is governed by a strong interaction between strain-specific traits, food matrix composition, and processing conditions, highlighting the need for a system-level approach rather than isolated technological solutions. Among the strategies currently explored, processing optimization and emerging technologies can effectively reduce technological stress, while encapsulation approaches offer improved protection but may introduce limitations related to cost, scalability, and sensory modifications. In parallel, stress adaptation enhances probiotic resilience and cross-protection under adverse conditions, while directed evolution can further improve these traits but may be limited by fitness costs, as well as regulatory and societal barriers.

Despite significant advances, most approaches have been evaluated under controlled laboratory or model food conditions. Their performance under industrially relevant food systems and extended storage periods is still not fully established. Future progress should focus on integrated, scalable solutions that balance technological feasibility, product quality, regulatory acceptance, and consumer perception, ensuring the development of robust and effective non-dairy probiotic foods.

Although this review provides a comprehensive overview of current strategies to improve probiotic viability in non-dairy foods, the available evidence remains highly heterogeneous due to differences in probiotic strains, food matrices and experimental conditions, limiting direct comparisons among studies. Moreover, industrial-scale validation and well-designed clinical trials remain scarce. Therefore, future research should focus on standardized experimental protocols and validation under real processing and consumption conditions.

## Data Availability

No new data were created or analyzed in this study. Data sharing is not applicable to this article.
